# VIN usual type—from the past to the future

**DOI:** 10.3332/ecancer.2015.531

**Published:** 2015-04-29

**Authors:** Mario Preti, Sarah Igidbashian, Silvano Costa, Paolo Cristoforoni, Luciano Mariani, Massimo Origoni, Maria T Sandri, Sara Boveri, Noemi Spolti, Laura Spinaci, Francesca Sanvito, Eleonora P Preti, Adriana Falasca, Gianluigi Radici, Leonardo Micheletti

**Affiliations:** 1 Preventive Gynecology Unit, European Institute of Oncology, Milano 20100, Italy; 2 M.F. Toniolo Hospital, Bologna 40100, Italy; 3 Villa Montallegro, Genova 16100, Italy; 4 HPV-Unit Gynecologic Oncology, Regina Elena National Cancer Institute of Rome, Rome 00100, Italy; 5 Department of Obstetrics and Gynecology, Vita Salute San Raffaele University School of Medicine, Milano 20100, Italy; 6 Division of Laboratory Medicine, European Institute of Oncology, Milano 20100, Italy; 7 Department of Obstetrics and Gynecology, University of Torino, Torino 10100, Italy; * The Italian HPV Study Group (IHSG)

**Keywords:** VIN, vulvar intraepithelial neoplasia, HPV infection

## Abstract

Usual vulvar intraepithelial neoplasia (uVIN) is the most common VIN type, generally related to a human papillomavirus (HPV) infection, predominantly type 16. The incidence of uVIN has been increasing over the last decades, and a bimodal peak is observed at the age of 40–44 and over 55 years.

Almost 40% of patients with uVIN have a past, concomitant or future HPV-associated lesion of the lower genital tract. HPV-related malignancies are associated with a persistent HPV infection. The host immune response is of crucial importance in determining clearance or persistence of both HPV infections and HPV-related VIN. About 60% of the patients present with symptoms. Clinical features of uVIN vary in site, number, size, shape, colour, and thickness of lesions. Multicentric disease is often present.

Most uVIN lesions are positive at immunohistochemistry to p16^ink4a^ and p14^arf^, but negative to p53.

Irrespective of surgical treatment used, uVIN recurrence rates are high. Positive margins do not predict the development of invasive disease and the need to re-excide the tissue around the scare remains to be demonstrated. Therefore, considering the low progression rate of uVIN and psycosexual sequelae, treatments should be as conservative as possible.

Medical treatments available are mainly based on immunotherapy to induce normalisation of immune cell count in uVIN. None are approved by the food and drug administration (FDA) for the treatment of uVIN. If medical treatment is performed, adequate biopsies are required to reduce the risk of unrecognised invasive disease. Some studies suggest that failure to respond to immunotherapy might be related to a local immunosuppressive microenvironment, but knowledge of the uVIN microenvironment is limited. Moreover, our knowledge of the potential mechanisms involved in the escape of HPV-induced lesions from the immune system has many gaps.

HPV vaccines have been demonstrated to be effective in preventing uVIN, with 94.9% efficacy in the HPV-naive population, while studies on therapeutic vaccines are limited. The low incidence of VIN requires large multicentre studies to determine the best way to manage affected patients and to investigate the immunological characteristics of the ‘vulvar microenviroment’ which leads to the persistence of HPV.

## Classification

The definition of vulvar intraepithelial neoplasia (VIN) refers to a proliferation of atypical basal cells in the vulvar epithelium. The acronym VIN was introduced at the beginning of the 1980s to define with a single term dysplastic lesions and carcinoma *in situ* of the vulva. Until then, these lesions were identified with different terms such as erythroplasia of Queyrat, Bowen’s disease, and hyperplastic dystrophy with atypia. The VIN classification was adopted in 1986 by the International Society for the Study of Vulvar Disease (ISSVD) including intraepithelial neoplasia of the squamous epithelium (divided into three grades based on the thickness of the epithelium affected by cellular atypia) and non-squamous VIN (Paget’s disease and vulvar melanoma *in situ*) [1].

There has been criticism of the 1986 ISSVD VIN classification. The first point of criticism was inclusion in the classification of Paget’s disease and vulvar melanoma *in situ* (not squamous intraepithelial neoplasia) that have different epidemiology, risk factors, and natural history compared to squamous VIN.

Other criticisms were as follows:

- Grading of VIN was similar to cervical intraepithelial neoplasia classification (CIN) despite the origin of VIN from a mature squamous skin/mucous epithelium and CIN from a transition epithelium.
- Clinical evidence that VIN1 does not represent a precancerous lesion but is just a sign of an HPV infection, a regressive alteration or an inflammatory disease.
- Reproducibility studies on the histopathological diagnosis of VIN that showed a very low reproducibility of VIN1 and a distinction between HPV-related VIN and non-HPV-related VIN

Therefore, in 2004, the ISSVD introduced a new classification for VIN on the basis of morphologic and pathogenetic criteria [2].

In this new classification, the diagnostic category of VIN 1 has been abolished, and histological changes previously included under the term VIN 1 are described as condyloma and/or HPV effects. The term VIN has been reserved only to the lesions previously classified as VIN 2 and 3 and differentiated VIN.

Three diagnostic terms were introduced to classify the specific pathogenetic VIN types as follows:

- VIN usual type (uVIN)
- VIN differentiated type (dVIN)
- VIN unclassified type (VIN NOS)

Usual type VIN (bowenoid, basaloid, and mixed) is the most common VIN type and is generally related to a human papillomavirus (HPV) 16 infection [3], and it occurs predominantly in younger women and tends to be multifocal.

Differentiated VIN is less common (2–10% of all reported VIN) and generally not related to HPV, it is mostly unifocal and usually found in older women with associated chronic dermatoses as lichen sclerosus.

## Epidemiology

The worldwide incidence of uVIN in young women has been increasing over recent decades [4–8]. From 1992 to 2005, the incidence of uVIN has almost doubled from 1.2/100,000 to 2.1/100,000, while the incidence of invasive vulvar cancer (IVC) remained stable. The increase in awareness of vulvar premalignant lesions may have led to the observed increase in VIN incidence, but also it may result in the removal of uVIN before these become invasive. A bimodal peak incidence of uVIN was observed at the age of 40–44 years and over 55 years [5, 7, 8]. A past, concomitant or future HPV-associated lesion of the lower genital tract is shown in 41% of patients with uVIN [8], with younger patients being at a higher risk of multifocal lesions. Van de Nieuwenhof *et al* showed that the risk of a subsequent diagnosis of vulvar squamous cell carcinoma (SCC) significantly increased with the age of diagnosis of uVIN, beginning with 2.7% for the age group 15–29 and increased to 8.5% for the group >75 years of age. In addition, they showed that the time between diagnosis of uVIN and SCC significantly shortened with increasing age, that is, 50 months for the age group of 15–29 years and 25 months for the age group of more than 75 years [8].

## Pathogenesis

The recent analysis of over 2,000 intraepithelial and invasive lesions of the vulva made by the HPV VVAP study group detected HPV-DNA in 86.7% of the VIN cases [9]. The low proportion of HPV-negative lesions correlates with the lower proportion of dVIN compared to uVIN. HPV 16 was the commonest genotype (77.3%) detected in uVIN, followed by HPV 33 (10.6%) and HPV18 (2.5%). In contrast to the high proportion of HPV-positive VIN, only 28.6% of IVC were HPV-DNA positive [9]. It is well known that IVC not related to HPV are more frequent than those related to HPV. These carcinomas are usually found in patients affected with lichen sclerosus or lichen planus and derived from dVIN.

The malignant potential of uVIN is difficult to assess being usually surgically removed to prevent invasive disease. A review of 3,322 treated and untreated VIN showed a progression to IVC in 9% of the untreated cases within 1–8 years, while in case of treated uVIN, the malignant potential was 3.3% [10]. The risk of future IVC in VIN-treated patients is considered to be around 3–4% [10–13].

Similar to the intraepithelial neoplasia found in IVC, the most common HPV type detected is HPV 16 (72.5%), followed by HPV 33 (6.5%) and HPV 18 (4.6%). While HPV 45 was almost not present in VIN, it was the fourth most common HPV (3.3%) in IVC showing a possible different contribution of this HPV genotype to HPV-associated invasion in the vulva [9].

HPV-related malignancies are associated with a persistent HPV infection. In 90% of cases, the immune system is capable of clearing a transient HPV infection within 2 years [14]. The host immune response is of crucial importance in determining clearance or persistence of both HPV infections and HPV-related VIN [14–19]. This is clearly seen in immune-compromised patients where the malignant potential of uVIN is 50-fold higher compared to the general population [20]. Failure of the immune system to produce an effective response to high-risk HPV is related to both viral persistence and various host factors [21]. HPV produces several proteins among which there are the oncoproteins E6 and E7. The longer the infection persists, the longer E6 and E7 can interfere with important control mechanisms of the cell cycle [22–26]. HPV E6 can lead to the dysfunction of the tumour suppressor gene p53 [27], while E7 can inactivate the retinoblastoma tumour suppressor gene (pRb), which leads to the overexpression of p16^ink4a^ and p14^arf^ and hyperproliferation of infected cells [24, 28]. Most uVIN lesions are positive at immunohistochemistry to p16^ink4a^ and p14^arf^, but negative to p53 [24–26, 29, 30]. The knowledge of the role of these defined tumour-specific antigens, E6 and E7, represents an excellent basis for the development of strategies aiming to reinforce the immune response to prevent HPV-related cancers.

## Clinical features and diagnosis

Screening tests for the diagnosis of VIN are not available: uVIN is diagnosed during the visual assessment of the vulva. To confirm the diagnosis, a biopsy of suspicious lesions should be performed using local anesthesia. The optimal biopsy is a punch or a small incision biopsy taken from the edge of the lesion with the inclusion of a piece of surrounding normal tissue [31].

uVIN can be asymptomatic, diagnosed during routine gynaecological visits, in patients with abnormal pap smear or positive HPV cervical test, or symptomatic in about 60% of the patients [10, 32].

**Figure 1.**
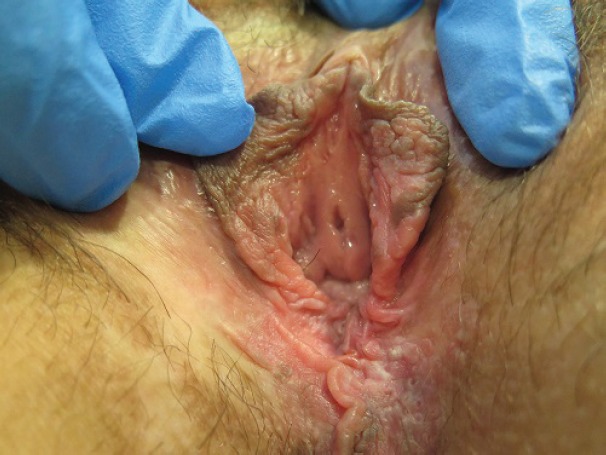
Twenty-nine-year-old patient with uVIN. Vulvar multifocal lesions: white and brown plaque on the inner surface of the left labium minus; white and brown papules on the perineum.

**Figure 2.**
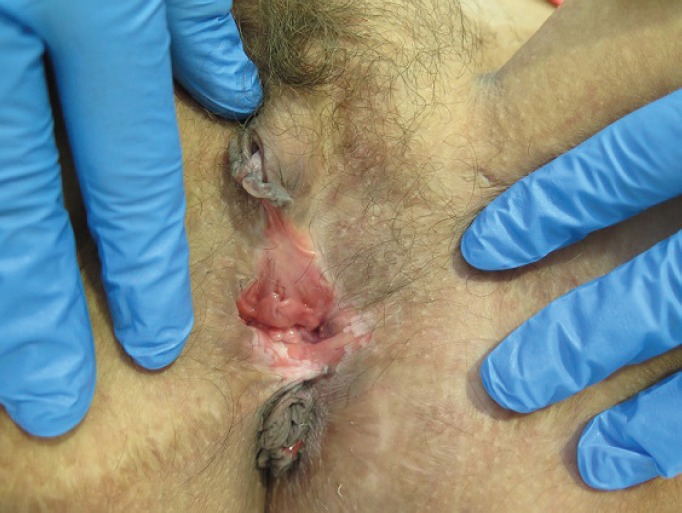
Thirty-eight-year-old patient with uVIN. Confluent white plaques on the posterior vaginal vestibule after partial simple vulvectomy for superficially invasive keratinising squamous cell carcinoma.

**Figure 3.**
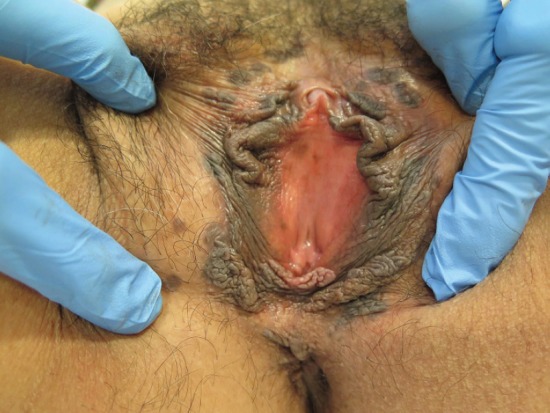
Thirty-nine-year-old patient with uVIN. Multifocal bilateral brown papules on the labia majora from the anterior part of vulva until the perineum.

**Figure 4.**
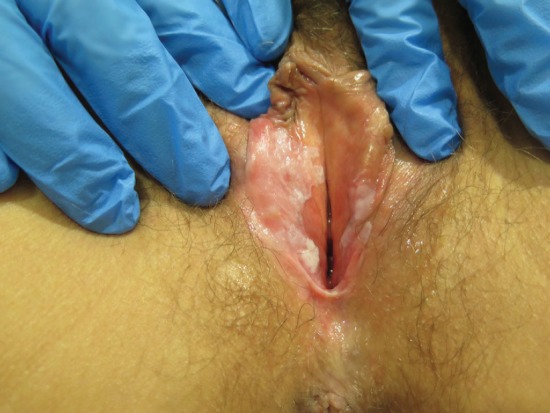
Fifty-four-year-old patient with uVIN. White papule on the inner surface of the posterior right labium minus in the context of confluent bilateral white macules on the vaginal vestibule.

**Figure 5.**
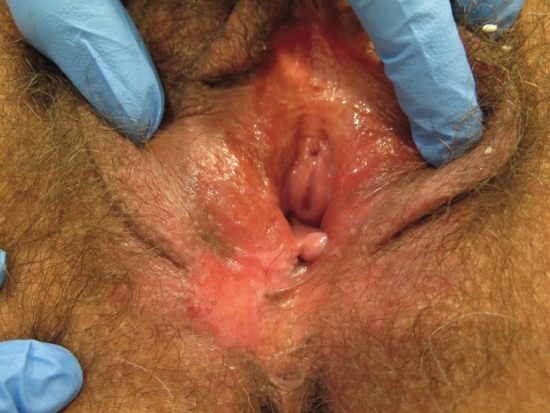
Fifty-nine-year-old patient with uVIN. Unifocal white, red, and brownish plaque on the right posterior part of the vulva, in the context of diffuse vulvar erythema, with excoriations caused by scratching.

The clinical features of uVIN vary in site anywhere on the vulva, number, size, shape, colour, and thickness of lesions. uVIN are usually multifocal, located around the introitus often involving the labia minora, and elevated. Lesions are sharply defined although the outline may be irregular or serpiginous [33] (Figures 1–5).

Multicentric disease (lesions of cervix, vagina, or anus) is often present in cases with uVIN, underlining the ‘HPV field effect’ that involves squamous epithelium from cervix to perianal area and the need of accurate examination in uVIN-affected patients. Therefore, a careful examination of all the vulva, perineum, perianal areas and comprehending also the cervix and vagina is mandatory.

## Treatment

The ideal approach to uVIN is to ensure the absence of stromal invasion, improve subjective symptoms, and reduce the risk of recurrence. Unfortunately, the surgical removal of the lesion represents, for most cases, the current standard approach to uVIN [33].

Indeed, no clinical appearance helps in distinguishing uVIN with stromal invasion from those without invasion, and in 12–17% of women undergoing VIN excision, a clinically unrecognised invasion is histologically diagnosed [11, 38–40]. In the past, extensive surgery has been performed to achieve this goal, leading in many cases to the disruption of both vulvar anatomy and function. More limited surgery is now being used in order to alter morphology as little as possible and to preserve vulvar function. Irrespective of the surgical treatment used recurrence rates are high [10, 11, 34]. Considering the low progression rate of uVIN and the young age of affected women treatments should be as conservative as possible with minimal effects on the psycosexual sphere and quality of life (QoL) of these women.

## Surgical treatment

Local excision removing all visible lesions can be performed with different techniques: scalpel, electrosurgery, or laser. All techniques seem to have a similar efficacy.

Surgery is still considered the treatment of choice, but surgeons should be as conservative as possible to reduce changes in the QoL of treated patients [33]. Optimal aesthetic and functional perineovulvar reconstruction is now considered as an integral part of the treatment of these lesions. Recurrence rate after excision ranges from 20% to 40% [12]. The presence of multifocal disease is associated with a higher incidence of recurrence [33, 34].

There are no definitive studies evaluating the safety margins in VIN resections, but recent papers demonstrated that positive margins do not predict the development of invasive disease [35]. In other words, when the pathological specimen describes involved margins and no stromal invasion is noted, for the most part of cases, it is enough to follow patient without re-exciding the tissue around the scare. Ongoing studies on adjuvant therapy with imiquimod in these cases are reported in next paragraph.

Carbon dioxide (CO_2_) laser excision yields a good cosmetic and functional result in experienced hands and gives a surgical specimen for histologic evaluation which is not present in case of CO_2_ laser vapourisation. Therefore, when CO_2_ laser vapourisation is planned, multiple biopsies should be performed to rule out invasion. Recurrence-free survival is lower in case of laser-vapourisation, probably because this technique is more often used in multifocal and multicentric lesions [11, 12, 36, 37].

## Medical treatment

Many medical treatments have been introduced to avoid or limit surgery in patients with uVIN. Most treatments lack of enough evidence because of the few number of subjects studied, of the different inclusion criteria, comparison groups or follow-up used in the studies [28]. Therefore, no medications are approved by the FDA for uVIN treatment.

Moreover, considering the high number of clinically unrecognised invasion found in the histology examination of VIN excisions, if surgical treatment is not performed, adequate biopsies are required to reduce the risk of unrecognised invasive disease.

Medical treatments available are mainly based on immunotherapy. The different forms of immunotherapies that have been developed and tested for the treatment of uVIN are aimed at overcoming the ‘inertia’ of the immune system in these patients.

### Imiquimod

Imiquimod, 1-(2-methylpropyl)-1H-imidazo[4, 5-c]quinolin-4-amine (also known as R-837 and S-26308), is a non-nucleoside heterocyclic amine that belongs to immune-response modifiers [41]. *In vivo* studies have demonstrated that imiquimod is a potent inducer of interferon (INF) α, tumour necrosis factor α, and interleukin-6 [42–44]. In animal models, imiquimod has demonstrated potent antiviral and antitumour effects [41].

Imiquimod 5% is licensed for the treatment of external genital warts, actinic keratosis, and basal cell carcinoma. Imiquimod 5% acts through a toll-like receptor (TLR7) inducing T cells activation and proinflammatory cytokines release [45, 46]. A thin layer of imiquimod 5% cream is applied on the lesion two or three times per week for a period of 12–16 weeks. Side effects are mainly local inflammatory reactions, itching, burning, and flu-like signs. In case of severe side effects, application can be reduced to once a week or stopped for a week.

The largest prospective, randomised, double-blind, placebo-controlled study analysed 52 cases, of which 35% had a complete response and 46% a partial response [47]. This study showed an effective long term efficacy of imiquimod, since VIN recurred in only one case of the complete respondents [48]. A recent meta-analysis that assessed 104 patients confirms the effectiveness of the treatment [49].

Imiquimod-induced clearance of HPV was associated with the normalisation of immune cell count in uVIN [48]. Many different pre-existing conditions can determine failure of patients’ responsiveness to immunotherapy: lesion size, lack of immune infiltration, CD4+ or CD8+ HPV-specific T-cell response, defects in HLA class I expression, IFNγ-associated response, genes of antigen presentation pathway, genes involved in T-cell migration and in chemo or cytokine production [21].

Imiquimod 5% has also been tested after surgery in comparison to surgery alone. The results in 80 patients demonstrated that during 5 years of follow-up, the overall complete response, the recurrence rate, and the disease-free time were similar in the two treatment options [50].

## Therapeutic vaccines

Therapeutic vaccines are designed to reinforce HPV-specific CD4+ and CD8+ T-cell responses in uVIN lesions [51]. Most vaccines elicit a specific immunity against HPV E6 and E7 proteins [52].

Two studies investigated the effectiveness of a combination of TA-HPV, a vaccinia virus encoding HPV16/18 E6/E7, and TA-CIN, an HPV 16 L2 E6/E7 fusion protein, in a total of 39 women with HPV 16 high-grade VIN. A reduction in lesion size was seen in 9 patients, while 25 remained stable and 5 progressed. This regimen proved to be immunogenic but no relation between induction of HPV 16-specific immunity and clinical outcome was observed [53, 54].

Kenter *et al* tested a synthetic long peptide (SLP) vaccine in 20 patients with HPV16-positive high-grade VIN. At 12 months of follow-up, 15 patients showed clinical response with a complete response in 9 cases. The complete response was maintained at 24 months of follow-up. In this trial, vaccine-induced T-cell responses were seen in all patients and complete responders had a significantly stronger response of IFNγ-associated proliferative CD4+ T cells and a broad response of CD8+ IFNγ T cells [55].

Small trials showed results with therapeutic vaccines; however, the costs for the development of vaccines are high, and at the moment, the focus is on preventive vaccines rather than therapies.

## Photodynamic therapy

Photodynamic therapy (PDT) uses a tumour-localising photosensitiser, 5-aminolevulinic acid, in combination with non-thermal light to generate tumour-directed cell death and induce local inflammation, which activates antigen-presenting cells (APCs) and induces effector T cells. Several non-randomised and uncontrolled studies were conducted on uVIN with response rates varying from 0% to 71%, with unifocal lesions responding better than multifocal, pigmented, and high-grade lesions [56–60]. Recurrence rate (around 48%) was similar to laser vapourisation and surgery [12]. Data on PDT are limited to small case series with variable response rates; patient tolerance of side effects, such as pain, could limit its use and costs are high.

## Known and unknown of immunological microenvironment

It has long been known that the adaptive immune response, in particular T cells, confer protection against HPV-induced diseases [61, 62]. Overall, HPV-induced diseases are associated with a lack of a strong HPV-specific CD4+ and CD8+ T cell [63]. The medical treatments available for uVIN are all associated with an increase in intralesional CD4+ and CD8+ T cells. Unfortunately, the available immunotherapeutic approaches often fail to induce clinical responses. Some studies suggest that failure to respond to immunotherapy might be related to a local immunosuppressive microenvironment, but knowledge on uVIN microenvironment is limited [19, 46, 48, 64–66]. Moreover, the knowledge of the potential mechanisms involved in the escape of HPV-induced lesions from the immune system has many gaps.

Winters *et al* found that initial not-responders to imiquimod seemed to be relatively refractory, probably because of their unfavourable local immune environment, in particular, the increased proportions of T regulatory cells (Tregs) [65]. Moreover, patients with lesions unresponsive to therapeutic vaccines showed a reduced systemic vaccine response but also increased number of lesions associated with immune suppressive Tregs [55, 66, 67]. Failure to cure some HPV-related premalignant and malignant lesions appears to result from an unfavourable balance in effector T cells and Tregs. Success of immunotherapies might need to address the means to alter this balance [68].

Spontaneous regression and clearance of HPV is associated with systemic HPV-specific CD4+ and CD8+ T-cell responses and normalisation of immune cell infiltration [62, 68]. The uVIN microenvironment is characterised by a dermal immune activity, with an influx of mature dendritic cells (DCs), natural killer cells (NK), and both CD4+ and CD8+ T cells, while the epidermis shows a decreased number of CD8+ T cells and increase in immature DCs and Langerhans cells (LCs) [19, 48, 56].

Moreover, van Esch *et al* showed an alteration in human leukocyte antigen (HLA) molecules not only in HPV-induced cancers but even in the premalignant stage. They detected a partial downregulation of HLA-class I in more than 70% of uVIN lesions and in 80% of HPV-related IVC. However, the downregulation of HLA class I expression in the majority of uVIN was not associated with the loss of heterozigosity (LOH) in contrast with the findings in IVC. This downregulation in uVIN was considered ‘soft wired’ since the expression can be restored applying some changes to the microenvironment, like stimulating with IFNγ. All patients with uVIN evaluated that showed a good clinical response to HPV 16 SLP vaccine had this partial downregulation in uVIN. In fact, the vaccine-induced HPV 16-specific type helper T cells infiltrating the lesion can provide IFNγ in the microenvironment [69].

Immunotherapy may strengthen the pre-existent activation of the local immune system, and immunologically active primary lesions may be more likely to respond to immunotherapy, also having a lower number of regulatory T cells subsets. Therefore, less immunologically active uVIN lesions, recurrent lesions and IVC may represent the harder challenge for immunotherapies [51].

An increased comprehension of the immunological environment that can be found in uVIN might help to better understand the conditions that favour the persistence of HPV and the development of uVIN and its potential oncogenic transformation to IVC. It might also help to define immunological markers that can predict responsiveness to immunotherapy.

## Prevention

HPV prophylactic vaccination has demonstrated to be effective in preventing uVIN. The vaccine efficacy against HPV 16- and/or HPV 18-related VIN is highest in the HPV-naive population, with a 94.9% efficacy [70]. Although prevention of IVC was not demonstrated, prevention of premalignant uVIN may anticipate a reduction of rates of HPV-related IVC [71].

HPV vaccines have also demonstrated a partial cross-protective efficacy against non-vaccine HPV types, such as HPV 31, 33, and HPV 45 [72–75]. This could lead to an even higher efficacy in reducing the incidence of uVIN and possibly IVC.

Moreover, studies on previous HPV vaccination in women treated for HPV-related diseases of the cervix, vulva, or vagina showed a reduction in subsequent cervical, vulvar, and vaginal intraepithelial neoplasia and genital warts [76].

An estabilished risk factor for vulvar cancer is smoking. Therefore, smoking should be discouraged [77].

## Conclusion

VIN is observed by a wide variety of medical specialists who should be aware of the crucial importance of careful vulvar examination, the malignant potential of the lesion, and the possibilities of conservative treatment depending on VIN localisation, size, focality, associated diseases, age, and psychological conditions of the patient. Long-term surveillance in all treated women is essential as removal of the lesion does not represent a complete prevention of invasive cancer.

The low incidence of VIN requires large multicentre studies to determine the best way to manage affected patients and to investigate the immunological characteristics of the ‘vulvar microenvironment’ that leads to the persistence of HPV.

## Conflicts of interest

The authors have no conflict of interest
